# The Use of Chinese Skullcap (*Scutellaria baicalensis*) and Its Extracts for Sustainable Animal Production

**DOI:** 10.3390/ani11041039

**Published:** 2021-04-07

**Authors:** Baishuang Yin, Wei Li, Hongyu Qin, Jinyan Yun, Xuezhao Sun

**Affiliations:** 1 College of Animal Science and Technology, Jilin Agricultural Science and Technology University, Jilin 132109, China; ybs3421@126.com (B.Y.); liweiscau@163.com (W.L.); qinhyvet@163.com (H.Q.); yunjinyan@hotmail.com (J.Y.); 2 The Innovation Centre of Ruminant Precision Nutrition and Smart and Ecological Farming, Jilin Agricultural Science and Technology University, Jilin 132109, China; 3 Jilin Inter-Regional Cooperation Centre for the Scientific and Technological Innovation of Ruminant Precision Nutrition and Smart and Ecological Farming, Jilin 132109, China

**Keywords:** *Scutellaria baicalensis*, extract, sustainable animal production, medicinal herb, feed

## Abstract

**Simple Summary:**

With the increasing pressure to address the problems of bacterial resistance and drug residues, medicinal herbs are gradually taking a more important role in animal production. *Scutellaria baicalensis* is a common and widely used Chinese medicinal herb. The main bioactive compounds in the plant are baicalein and baicalin. These compounds have many biological functions including anti-oxidation, antipyretic, analgesic, anti-inflammatory, antiallergic, antimicrobial, immunomodulatory, and antitumor effects. *S. baicalensis* and its extracts can effectively promote animal growth, improve the production performance of dairy cows, reduce the stress and inflammatory response, and have effective therapeutic effects on diseases caused by bacteria, viruses, and other pathogenic microorganisms. This paper summarizes the biological function of *S. baicalensis* and its application in sustainable animal production to provide a reference for future application of *S. baicalensis* and other medicinal herbs in animal production and disease treatment.

**Abstract:**

Drugs have been widely adopted in animal production. However, drug residues and bacterial resistance are a worldwide issue, and thus the most important organizations (FAO, USDA, EU, and EFSA) have limited or banned the use of some drugs and the use of antibiotics as growth promoters. Natural products such as medicinal herbs are unlikely to cause bacterial resistance and have no chemical residues. With these advantages, medicinal herbs have long been used to treat animal diseases and improve animal performance. In recent years, there has been an increasing interest in the study of medicinal herbs. *S. baicalensis* is a herb with a high medicinal value. The main active compounds are baicalin and baicalein. They may act as antipyretic, analgesic, anti-inflammatory, antiallergenic, antimicrobial, and antitumor agents. They also possess characteristics of being safe, purely natural, and not prone to drug resistance. *S. baicalensis* and its extracts can effectively promote the production performance of livestock and treat many animal diseases, such as mastitis. In this review, we summarize the active compounds, biological functions, and applications of *S. baicalensis* in the production of livestock and provide a guideline for the application of natural medicines in the production and treatment of diseases.

## 1. Introduction

Food from animal sources provides the human body with protein, fat, minerals, vitamin A, B vitamins, and other nutrients, thus giving animal products an important role in the food supply worldwide. Improving the health and production of food animals would also benefit human health. Manufactured chemical and antibiotic feed additives are widely used in animal husbandry, resulting in growing problems with antibiotic resistance and chemical residues, which are becoming limiting factors in animal husbandry [1,2,3,4]. Concerns over these issues have prompted various organizations and countries in the world to introduce policies for banning or restricting the use of chemicals and antibiotics as feed additives [5]. The “one health” approach is used worldwide [6,7]. Future actions and the World Health Organization action plan against antimicrobial resistance are based on best practices in implementing and monitoring the “one health” plans, supporting novel solutions to prevent and treat infections, thereby increasing the efforts in terms of combating antimicrobial resistance and related risks worldwide [8]. Medicinal herbs, with their unique advantages as natural products, might be one of the solutions to these issues in animal production.

Medicinal herbs and their extracts are natural, efficient with few side effects, and have minimal risk of inducing bacterial resistance [9]. At present, the application of medicinal herbs and their extracts in animal production is still at the emerging stage. It has been proven that medicinal herbs can effectively improve the utilization of feed protein, and the performance of growth and reproduction in animals [10,11], modulate rumen fermentation towards high efficiency [12,13,14], increase feed intake and digestibility by affecting feed intake behaviors and the secretion of digestive enzymes [14], alleviate the effects of stress by enhancing antioxidant ability [15,16], and consequently improve the health status of animals. Furthermore, the antimicrobial effects of medicinal herbs can also be used for the prevention and treatment of diseases. Thus, medicinal herbs as alternative natural feed additives to replace chemicals and antibiotics have attracted much attention [17].

*Scutellaria baicalensis* is an herb that has roots of high medicinal value. It has the functions of clearing heat and detoxification, purging fire and drying dampness, improving fertility, and hemostasis. It is also widely used in clinical practice. This paper reviews the research progress on the use of *S. baicalensis* and its extracts in the sustainable production and health of animals. The biological functions of *S. baicalensis* and its extracts and their applications in animal production and disease prevention are briefly described to form a reference for the future research and application of herbal preparations in animal production.

## 2. *Scutellaria baicalensis* and Its Active Components

*Scutellaria baicalensis*, also known as Chinese skullcap or Baikal skullcap, is a perennial herb of the family Lamiaceae. It mainly grows at an altitude of 60–2000 m in sandy soil on sunny slope land. The plant is natively distributed in East Asia and widely cultivated in European and American countries. China is the main producer of *S. baicalensis* for medicinal use [18]. The dry roots of the plant are often used as medicine. The chemical components of *S. baicalensis* roots are mainly flavonoids, anthraquinones, lignin, organic acids, volatile oils, and other compounds. Hereafter, *S. baicalensis* roots are sometimes referred to as *S. baicalensis.* The characteristic components of *S. baicalensis* are baicalin, baicalein, scutellarin, and scutellarin. Baicalin is a monomer active component extracted from the roots of *S. baicalensis*. Baicalein is the aglycon form of baicalin, which is a typical component of *S. baicalensis* [19].

**Figure 1:**
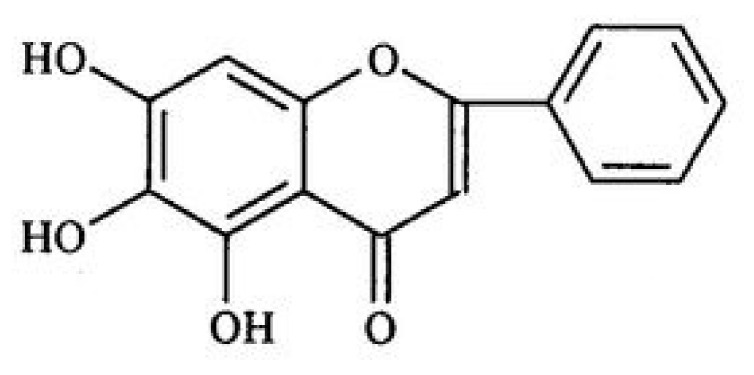
Molecular structure of baicalein. The IUPAC name of baicalein is 5,6,7-Trihydroxy-2-phenylchromen-4-one, or also known as 5,6,7-trihydroxyflavone.

**Figure 2:**
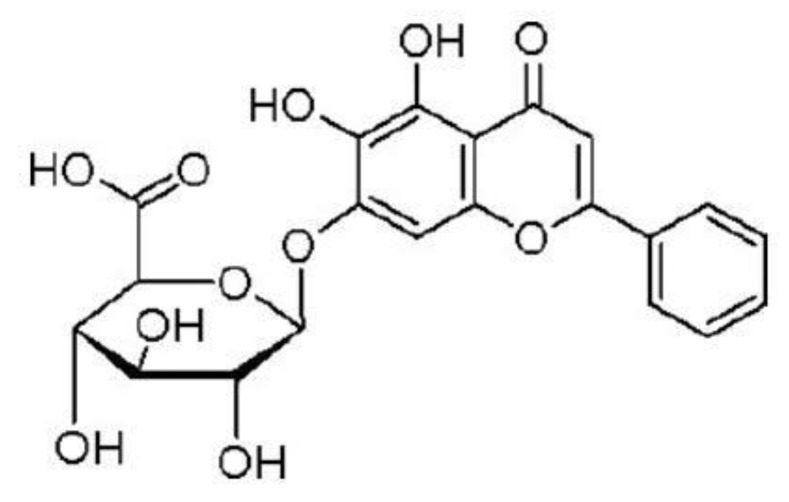
Molecular structure of baicalin. The IUPAC name of baicalin is (2S,3S,4S,5R,6S)-6-(5,6-dihydroxy-4-oxo-2-phenylchromen-7-yl)oxy-3,4,5-trihydroxy-tetrahydropyran-2-carboxylic acid, or also known as baicalein 7-*O*-glucuronide.

Baicalein and baicalin are the main active compounds of *S. baicalensis*, which are flavonoids. Baicalin is the glucuronide of baicalein [19]. The molecular formula of baicalein is C_15_H_10_O_5_ with a relative molecular mass of 270.24, and baicalein can be crystallized as yellow needles and is easily soluble in alcohol, acetone, and slightly soluble in chloroform. The chemical structure is shown in Figure 1. The molecular formula of baicalin is C_21_H_18_O_11_, with a relative molecular weight of 446.36. Pure baicalin exists as light yellow crystal needles and is soluble in hot acetic acid, insoluble in acetone, methanol, and ethanol, and almost insoluble in water. The molecular structure is shown in Figure 2.

## 3. Biological Functions of *S. baicalensis*

*S. baicalensis* has a long history as a medicinal herb due to its extensive biological and pharmacological activities. Its use in treating lung and liver diseases was first recorded in Sheng Nong’s herbal classic during the Han Dynasty in China. *S. baicalensis* has a cooling effect and is bitter in taste. It has antimicrobial, anti-inflammatory, antipyretic, and analgesic effects and improves animal growth performance (Table 1). It plays an important role in livestock and poultry production [20]. Baicalin, as the most abundant flavonoid in the roots of *S. baicalensis*, is stable in acidic environments and organic solvents but unstable in alkaline environments and in the plant. The absorption rate of baicalin is low via oral administration. However, baicalein formed from enzymatic hydrolysis of baicalin in the intestinal tract is easily absorbed into the blood and then is chemically converted into baicalin and other metabolites in the liver showing biological activities [21,22].

### 3.1. Antioxidation

Flavonoids have significant antioxidant activity. There are two antioxidant mechanisms of flavonoids. One is a direct hydrogen pumping reaction mechanism. The antioxidant molecule loses the phenolic hydroxyl hydrogen atom to generate phenolic oxygen radicals, and its antioxidant activity depends on the ease of phenolic hydroxyl O-H bond breaking. The other one is a single electron transfer mechanism. The antioxidant transfers one electron to the reactive oxygen radicals to generate the flavonoid radical cation [67]. The A-ring of baicalin contains an o-diphenol structure, whereas the molecular structure of baicalein contains three hydroxyl groups [68]. The key to baicalin’s antioxidant mechanism is the regulation of nuclear factor-erythroid 2-p45 derived factor 2 (Nrf2). Nrf2 is the core transcription factor that regulates the cellular oxidative response. It can induce the activity of the antioxidative reactive protein (Ares) and thus inhibit the formation of oxidative free radicals (ROS), consequently reducing oxidative stress to keep the stability of the intracellular environment [25]. Baicalein can scavenge free radicals by decarboxylation in the body to regulate oxidative stress. Baicalein has a strong scavenging effect on free radicals such as alkane peroxide and superoxide anion [45].

Gao et al. [23] detected free radicals using the electron spin resonance (ESR) technology and found that baicalin and baicalein have a strong scavenging effect on hydroxyl radicals, 2,2-diphenyl-1-picrylhydrazyl (DPPH) radicals, and alkyl peroxide radicals. In addition, baicalein can inhibit xanthine oxidase activity and thus inhibit the production of oxygen free radicals during its metabolism [19]. Peroxides (such as hydrogen peroxide) can induce neuronal cell injury. Baicalin and baicalein have significant protective effects on the oxidative injury of human neuronal cells induced by H_2_O_2_, and baicalein has stronger antioxidant activity than baicalin [43]. The free radical scavenging and antioxidant effects of baicalin and baicalein can be used to effectively treat free radical and oxidative stress-related diseases.

To protect the heart, baicalein can concentration-dependently reduce hypoxia reoxygenation induced myocardial death and apoptosis. Further studies revealed that the cardioprotective effects of baicalein were mediated through μ- and δ-, but not κ-opioid receptors and their associated signal transduction pathways, such as protein kinase C and ATP-sensitive potassium (K_ATP_) channels [44]. Baicalin can effectively attenuate oxidative stress and apoptosis by activating the Nrf2 signaling pathway, thereby protecting the thymus from structural and functional damage mediated by mycoplasma infection [24].

### 3.2. Antipyretic and Analgesic Effects

**Figure 3:**
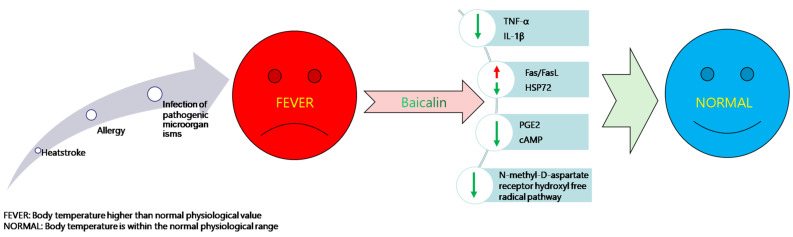
Schematic diagram of the antipyretic mechanism of baicalin. FEVER: Body temperature is higher than the normal physiological value. NORMAL: Body temperature is within the normal physiological range.

Baicalin has a significant antipyretic effect (Figure 3), and the mechanism underlying the effect may be related to the reduction of the contents of tumor necrosis factor-α (TNF-α) and interleukin-1β (IL-1β) in a significant dose-dependent manner [27]. The antipyretic effect of baicalin is via inhibiting the hydroxyl radical pathway of the N-methyl-D-aspartate receptor in the hypothalamus and accumulating TNF-α during fever. Studies have shown that baicalin can play an antipyretic role by reducing the concentration of prostaglandin E2 (PGE2) and cyclic adenosine monophosphate (cAMP) in the hypothalamus [28]. Other studies have found that baicalin reduces heat-stress-induced apoptosis by regulating the Fas/FasL pathway and upregulating heat shock protein 72 (HSP72) expression in bovine testicular Sertoli cells [29]. In addition to its antipyretic effect, baicalin also has a substantial analgesic effect [30]. It is found that baicalin has anti-inflammatory and analgesic effects in the studies on the evaluation of its effects on inflammation, pain, and edema [31]. Pain-related animal models were used to evaluate the analgesic activity of UP446 (a standard bioflavonoid component of baicalin and catechol). It was found that the pain sensitivity of hyperalgesic animals induced with carrageenan pretreatment reduced by 39.5% after an oral administration at 150 mg UP446/kg body weight. In the writhing test and formalin test, a single dose of UP446 orally administered at 100 mg/kg body weight showed 58% and 72% inhibition of pain sensitivity, respectively [32].

### 3.3. Anti-Inflammatory and Antiallergic Effects

*S. baicalensis* and its extracts have a strong anti-inflammatory function, participate in the regulation of a variety of inflammatory factors, and also have cartilage protection effects [52]. Baicalin and baicalein treat skin diseases by regulating enzymes and pro-inflammatory factors such as interleukin-6, oxygenase-1, TNF-α, and cell adhesion factors [33,46,53]. In vitro experiments showed that baicalin has a protective effect against lipopolysaccharide (LPS)-induced inflammatory injury in mammary epithelial cells and can play an anti-inflammatory role by inhibiting nuclear factor kappa-B (NF-κB) activation and mitogen-activated protein kinase (p38) phosphorylation, and upregulating HSP72 to alleviate LPS-induced inflammation and cell apoptosis in mammary epithelial cells [69,70]. A study on rats found that baicalein can inhibit the release of interleukin-8 and the synthesis of cyclooxygenase-2 and increase the formation of heat shock protein 70 to improve the anti-inflammatory ability of the body, consequently blocking the inflammatory injury caused by inflammatory factors [71]. Baicalin can inhibit the production of inflammatory factors TNF-α, IL-1β, Interleukin-6 (IL-6), Interleukin-17 (IL-17), matrix metalloprotein-9 (MMP-9), and regulate NF-κB signaling pathway to have anti-inflammatory effects [72].

Baicalein and baicalin can also inhibit vascular permeability, the adsorption and migration ability of leukocytes, and inhibit the production and release of inflammatory mediators, providing a reference and theoretical basis for the development of drugs to treat cardiovascular-related diseases [73]. In addition, baicalin can reduce inflammation and edema as a dual inhibitor of cyclooxygenase and 5-lipoxygenase [34]. *S. baicalensis* exerts antiallergic effects mainly by inhibiting the mast cell degranulation process and inhibiting the release of the histamine slow-reacting substance of anaphylaxis (SRS-A). This may alleviate the itching, gastrointestinal contraction, and other symptoms caused by type I, II, and IV allergic reactions in animals, without apparent side-effects [59,60,61,62,63,64,65].

### 3.4. Antimicrobial Effect

The plant extract of *S. baicalensis* has a broad-spectrum inhibitory effect on the growth of bacteria, including mycoplasma and spirochetes type bacteria, fungi, and viruses. Baicalin and baicalein are the active compounds that inhibit the growth of bacteria by destroying the nucleic acid formation of bacteria and altering the energy metabolism of bacteria, as well as inhibiting the formation of biofilms of bacteria such as *Klebsiella pneumoniae* and *Pseudomonas aeruginosa* [74]. In experiments evaluating the inhibitory effect of baicalin on milk-derived *Escherichia coli*, it was found that baicalin had an inhibitory effect on *E. coli* in vitro [35]. After the use of baicalin, the sensitivity of most strains to other antimicrobial agents was enhanced [36].

In addition, *S. baicalensis* and its extract have a bactericidal effect on *Helicobacter pylori*, *Staphylococcus aureus*, and other pathogenic bacteria [55,56]. Baicalin in conjunction with penicillin and ciprofloxacin has a synergistic effect on the treatment of penicillin-resistant *S. aureus* and methicillin-resistant *S. aureus* (MRSA) [37,38]. Baicalein can prevent the formation of bacterial biofilms and disrupt the biofilms and consequently reduce the production of staphylococcal enterotoxin A and α-hemolysin, thereby inhibiting the growth of *S. aureus* [47]. In addition, baicalein can reduce the pathogenic ability of bacteria such as *S. aureus* and *E. coli* by disrupting their cell wall integrity, reducing bacterial enzymatic activities, and inhibiting bacterial energy production and nucleotide synthesis [48]. It was found that the extract of *S. baicalensis* has significant antifungal effects, such as *Candida albicans*, *Aspergillus fumigatus*, *Hydramycetes*, etc. Baicalein and wogonin have strong antifungal activities, which may induce programmed apoptosis of the fungal cells via excessive production of reactive oxygen species [49]. In addition, the combination of baicalein with fluconazole and other antifungal drugs can more effectively treat fungal infections such as *Candida* [50]. The extract of *S. baicalensis* has an in vitro inhibitory effect on tick-borne encephalitis virus via direct inhibition of the adsorption and intracellular replication of tick-borne encephalitis virus [57]. *S. baicalensis* also has marked therapeutic effects on viral diseases such as influenza, infectious bronchitis, and viral diarrhea [54,58]. Relevant studies have shown that baicalin has a polyphenolic hydroxyl structure and prevents glyoxal-induced cystatin aggregation by affecting its aggregation process, which opens a new way for the treatment of protein misfolding diseases [75]. In addition, baicalin extract has potential applications in the fields of anti-fibrosis, anti-cancer, anti-aging, anti-depression, and immune regulation [76,77,78,79,80].

### 3.5. Antitumor Activity

Baicalin is able to induce the apoptosis of tumor cells. Baicalin could promote apoptosis in pancreatic cancer cells (SW1990 cell line) via dose-dependent upregulation of the expression of mitochondrial Bax (Bcl2-associated Xprotein) and cleavage-type enzymes caspase-3, and p53, consequently significantly decreasing B-cell lymphoma/lymphoma 2 (Bcl-2) protein levels, possibly activating c-Jun N-terminal kinase/forkhead box protein O1/Bcl-2 interacting mediator of cell death (JNK/Foxo1/BIM) pathway [39]. Baicalin can induce apoptosis in hepatoma cell lines HepG2 and SMMC-7721 cells [40]. It can also induce colon cancer cell apoptosis through microRNA-217/dickkopf (miR-217/DKK1)-mediated inhibition of the wingless-related integration (Wnt) signaling pathway [41]. Moreover, the combination of baicalin with drugs such as hexamethylene bis-acetamide (HMBA) showed promising efficacy against leukemia [42].

Baicalin can inhibit tumor invasion and metastasis. Baicalin effectively inhibits the invasion, migration, and adhesion abilities of multiple tumors by inhibiting the expression levels and activities of mitochondrial membrane potential 2 (MMP2) and mitochondrial membrane potential 9 (MMP9) [81]. Phosphorylation of AMP-activated protein kinase (AMPK) leads to mitochondrial membrane potential (MMP) expression and promotes tumor invasion and metastasis [82].

Baicalin inhibits non-small cell lung cancer migration and invasion by down-regulating MMP expression through activation of the mammalian target of rapamycin (mTOR) and silencing information regulator1/AMP-activated protein kinase (SIRT1/AMPK) signaling pathways [83]. In addition, baicalin also has the ability to induce cancer cell cycle arrest [84,85], overcome the drug resistance of tumor cells [86], and modulate tumor-associated inflammatory microenvironment [70,87].

Baicalein inhibits tumor cell development by modulating different metabolic signaling pathways, as well as decreasing tumor growth and metastasis rates, significantly decreasing CD31 (an endothelial cell marker) and α-smooth muscle actin (α-SMA, a parietal cell marker) expression and inducing cell death in tumor tissues [51].

Angiogenesis is the key process to promote cancer, and *S. baicalensis* has anti-angiogenesis activity in vitro. Liu et al. [66] evaluated the potential of baicalin and baicalein as antiangiogenic agents through the analysis of chicken chorioallantoic membrane (CAM) and the culture of human umbilical vein endothelial cells (HUVEC) in vitro. The study showed that baicalin and baicalein had the potential of antiangiogenesis and inhibited the migration of endothelial cells and the differentiation of endothelial cells into tubular branch networks in a dose-dependent manner [66]. Wang et al. [88] also determined the activity of angiogenesis through the proliferation of blood vessels on the chicken CAM model and cultured bovine aortic endothelial cells (BAEC). The study indicated that *S. baicalensis* could significantly inhibit the activity of angiogenesis.

Wogonoside is an active component of *S. baicalensis*. It can inhibit the migration and angiogenesis of HUVEC stimulated by LPS, as well as the microvascular sprouting of rat aortic rings in vitro. It may also have potential therapeutic value for diseases related to inflammation and angiogenesis [89]. Oroxylin A (one of the active components of *S. baicalensis* and one of the metabolites of baicalin in vivo) can also inhibit LPS-induced angiogenesis and may affect the lipopolysaccharide/Toll-like receptor 4 (LPS/TLR4) signaling pathway [90]. Wogonin also inhibits H_2_O_2_-induced angiogenesis by inhibiting the phosphatidylinositol 3-kinase/protein kinase B (PI3K/Akt) and NF-κB signaling pathway [91].

## 4. Application of *S. baicalensis* in Sustainable Animal Production for Better Performance

Medicinal herb extracts have been used as feed additives, veterinary medicines, and environment-friendly disinfectants [92] with support from the public due to their natural, safe, and effective characteristics. As an important medicinal herb, *S. baicalensis* can enhance the immunity of the body, reduce allergic reactions, protect the liver, and treat the body’s peroxidative reactions [19]. *S. baicalensis* could play an important role in the improvement of animal growth and production performance. Table 2 summarizes the main effects of *S. baicalensis* in animal production.

### 4.1. Poultry

Zhou et al. [93] evaluated the effects of baicalein on growth performance, immunity, and antioxidant activity of broilers at doses of 100 and 200 mg/kg diet. Compared with the basal diet, the baicalein-supplemented diet had no significant effect on the average daily feed intake but significantly increased body weight, average daily gain, and feed conversion efficiency of broilers at the age of 21–42 days and 7–42 days. The best growth performance was observed at the dose of 200 mg/kg diet. Compared with the control group, baicalein significantly increased CD3+/CD4+ and CD3+/CD8+ ratio, interferon γ (IFN-γ) concentration, anti-IB antibody titer, and spleen index. Total cholesterol, triglyceride, and low-density lipoprotein cholesterol were significantly decreased after baicalein intake compared with basal diet, while superoxide dismutase (SOD), glutathione peroxidase (GSH-Px), and catalase (CAT) activities in serum were increased with baicalein supplementation, and total antioxidant capacity (T-AOC) activity, total superoxide dismutase (T-SOD), and GSH-Px levels in liver tissues were significantly increased, while the intake of baicalein was significantly decreased. Malondialdehyde levels in serum and meat tissues were reduced. Baicalein can be used as an effective natural feed additive in broiler diets, and a 100–200 mg/kg diet is evaluated as the optimal dose. Króliczewska et al. [94] evaluated the effect of the roots of *S. baicalensis* on the production performance of broilers and found that the addition of *S. baicalensis* roots to the diet (5–15 mg/kg diet) was able to increase broiler body weight and feed conversion efficiency but had no effects on the quality and chemical composition of broiler leg muscles.

The addition of *S. baicalensis* extracts to the drinking water of turkeys (0.009, 0.018, and 0.036 mL/kg bodyweight) was able to change the concentrations of sodium, potassium, calcium, magnesium, copper, zinc, and iron in plasma, showing an upward trend in the concentrations of calcium and magnesium and a downward trend in the concentration of sodium, potassium, copper, zinc, and iron [95]. In addition, the dietary supplementation of *S. baicalensis* extracts to laying hens at a dose of 5 g/kg diet effectively increased the weight of the eggs, decreased the microbial content in the cecum, reduced the amount of propylene glycol, and delayed lipid oxidation in the eggs [96]. Fermented medicinal plants (*Gynura procumbens*, *Rehmannia glutinosa*, and *S. baicalensis*) at doses of 0.5–2 g/kg diet can be used as an alternative to reduce the use of antimicrobial agents in broilers for improved production performance [108].

Medicinal herbs are often applied in the form of a composite preparation after the assessment of compatibility. Varmuzova et al. [97] found that Curcuma (*Curcuma longa*) extract alone was not enough to reduce intestinal inflammation caused by heat stress. However, the mixture of *C. longa* and *S. baicalensis* plant extracts as feed additives reduced intestinal inflammation caused by a high air temperature or by *Salmonella enteritidis*, reduced the counts of *Salmonella* in cecum-midgut, and had no negative effects on body weight or humoral immune response. Using the 16S rRNA sequencing technique, it was found that the dietary supplementation of the two plant extracts had no effects on microbial diversity. However, if the plant extract supplements are provided to chickens infected with *S. enteritidis*, *Enterococcus faecalis*, and *Lactobacillus* spp., the bacterial genera with known positive effects on intestinal health are actively selected. Therefore, the supplementation of chicken feed with *Curcuma* and *Scutellaria* plant extracts can be used in poultry production to effectively reduce intestinal inflammation and improve chicken production performance [97]. Liu and Kim [109] found that the addition of *S. baicalensis* and *Lonicera japonica* extracts to the feed at a dose of 0.25–0.5 g/kg diet alleviated the detrimental effects of seasonal heat stress on the production performance of laying hens. Lv et al. [98] found that the dietary supplementation of *S. baicalensis* and *L. japonica* extracts could improve the growth performance of broilers, promote the development of immune organs, and improve the antioxidant function. Their results showed that the addition of 500 mg plant extracts/kg of diet could increase the average daily weight gain at the age of 21–42 days and 1–42 days, increase feed conversion efficiency, increase the thymus and bursa index of 42-day-old broilers, increase the activity of serum catalase, and decrease the level of malondialdehyde in 21- and 42-day-old broilers.

Cheng et al. [110] studied the anti-inflammatory effect of baicalin on LPS-induced chicken liver inflammation and its molecular mechanism. Histopathological changes, serum biochemical analysis, nitric oxide (NO) level, and myeloperoxidase activity showed that baicalin pretreatment alleviated LPS-induced liver inflammation. ELISA and qPCR analysis showed that baicalin dose-dependently inhibited the formation of IL-1β, IL-6, and TNF-α. In addition, baicalin significantly decreased the mRNA expression of inducible nitric oxide synthase (iNOS) and cyclooxygenase-2 (COX-2). Molecular studies showed that baicalin pretreatment inhibited TLR4 expression and the activation of the NF-kB signaling pathway. Baicalin pretreatment down-regulated TLR4 expression and inhibited NF-kB activation, thus had a protective effect on chicken liver from LPS-induced inflammation. Ishfaq et al. [111] also showed that baicalin treatment could effectively prevent *Mycoplasma gallinarum*-induced inflammation, apoptosis, and energy metabolism dysfunction, providing a basis for new therapeutic targets to control *M. gallinarum* infection. However, Króliczewska et al. [112] found that excessive supplementation of *S. baicalensis* roots (5–15 g/kg diet) may cause immunosuppression and may have a negative impact on the development of immune organs. *S. baicalensis* roots inhibited the formation of radially segmented nuclei, showing anti-metastatic properties and phagocytosis of chicken heterophils.

Dietary supplementation of *S. baicalensis* extracts improves growth performance, blood parameters, nutrient digestibility, and meat quality of broilers [113]. The combined use of *S. baicalensis* extract and Zn in the feed not only improved the quality of broilers but also showed antioxidant capacity [114]. Liang et al. [99] examined the effects of baicalin on the growth performance and intestinal bacterial community of broiler chickens at different doses supplemented to the basal diet and found that the average body weight of broilers in all treatment groups was increased compared with the control group. Among different treatments, the dose of 10 mg baicalein/kg diet had the highest body weight and the supplementation of 5 mg/kg baicalein to the diet increased the average daily feed intake. Compared with the control group, the number of *E. coli* and *Salmonellas* were decreased and the number of *Lactobacillus* and *Bifidobacterium* were increased in all experimental groups. The addition of an appropriate dose (10 mg/kg diet) of baicalein in the diet could promote the growth of broilers and modulate the intestinal microbial community. Wang et al. [115] studied the potential alleviative effect of combined plant extracts of *L. japonica* and *S. baicalensis* (active ingredients are chlorogenic acid and baicalin) on intestinal damage and bacterial dysbiosis caused by *Salmonella pullorum*. It was found that this preparation could effectively alleviate the intestinal damage and the loss of animal performance caused by *S. pullorum*, for which the regulation of intestinal microbial composition by the plant extracts is an important mechanism of action.

### 4.2. Swine

Li and Diao [100] found that the addition of fermented *S. baicalensis* to the diet could enhance the appetite of weaned piglets at the age of 28 days, increase the average daily intake, reduce the feed-to-weight ratio and diarrhea rate, and improve the feed conversion rate. Among the different doses, a dose of 1.5 g/kg diet had the best effect on daily intake. With this dosage, feed intake and daily weight gain were increased by 13% and 33%, and the feed-to-weight ratio and diarrhea rate decreased by 15% and 31% compared with the control group, respectively.

*S. baicalensis* and its extracts can effectively improve the growth performance and manipulate the intestinal microflora of pigs. Zhao et al. [101] evaluated the dietary supplementation of fermented medicinal plants (FMP) consisting of *Gynura procumbens*, *R. glutinosa* and *S. baicalensis* in weaned piglets. It was found that FMP additives significantly increased the average daily weight gain, average daily feed intake, the ratio of body weight to feed, and the apparent total tract digestibility of dry matter, nitrogen, and gross energy, and decreased the concentrations of ammonia, total thiol, and hydrogen sulfide. In addition, the diarrhea rate of piglets was reduced with the addition of FMP. Thus, FMP can be used to improve growth performance and nutrient digestibility, mitigate harmful gas emissions from feces, and reduce the early diarrhea rate of weaned piglets.

The addition of FMP containing *S. baicalensis* to the diet of fattening pigs was able to improve body weight gain, feed conversion efficiency, and digestibility and mitigate harmful gas emissions [116,117]. The combination of *S. baicalensis*, *Gardenia jasminoides,* and lactic acid bacteria can accelerate the clearance time of bacteria in feces, improve the immunity of infected pigs, and regulate the enzymatic activity of intestinal microorganisms in order to convert herbal compounds into active compounds [102]. Therefore, this mixture as a feed additive can be a potential preventive agent for *Salmonella* infection.

The addition of *S. baicalensis* extract to the diet at 1000 mg/kg diet can effectively alleviate diarrhea in weaned piglets and may reduce the expression of inflammatory cytokines by inhibiting the nuclear factor kappa-B/mitogen activated protein kinase (NF-κB/p38) signaling pathway, thereby improving intestinal health [103]. Baicalin in combination with aluminum can reduce piglet diarrhea. Genomic analysis indicated that the baicalin aluminum complex preparation could modulate the gut microbiota of diarrheal piglets and was associated with flagellar assembly, bacterial chemotaxis, lipopolysaccharide biosynthesis, ATP binding cassette transporters, biosynthesis of amino acids, and phosphotransferase systems [104]. The addition of mixed medicinal herbs containing *S. baicalensis* to the diet of pregnant and lactating sows can lead to decreased weight loss after delivery and can improve litter performance [107].

In addition, baicalin improves parthenogenetically activated and in vitro fertilized pig embryos by inhibiting the production and apoptosis of reactive oxygen species, regulating mitochondrial activity, and activating sonic hedgehog signaling [118].

### 4.3. Ruminants

*S. baicalensis* extracts can alter the microbial flora in the rumen of ruminants, thereby promoting the fermentation of forage in the rumen [119]. *S. baicalensis* has evident antipyretic effects and has been incorporated into many medicinal herb preparations [120], and thus has great potential for reducing the impact of heat stress in intensive and large-scale ruminants farming. Baicalin can be used as an anti-apoptotic agent to alleviate heat stress-induced apoptosis. Guo et al. [29] examined the effects of baicalin on heat stress-induced apoptosis of bovine Sertoli cells using flow cytometry and found that pretreatment with baicalin at 1, 10, and 20 μg/mL significantly reduced apoptosis induced by heat stress at 43 °C for 1 h. There was a dose-dependent relationship between baicalin concentration and the rate of cell survival.

Systemic inflammation is more common in early lactation dairy cows and can result in a decline in milk production. *S. baicalensis* contains flavonoids with anti-inflammatory and antioxidant effects. Olagaray et al. [26] incorporated *S. baicalensis* extracts into pelleted feed and provided them to lactating Holstein dairy cows at a dose of 10 g extracts/d (containing 3.3 g of flavonoid baicalin/d), which resulted in a reduced incidence of mastitis. A long-term supplementation (60 days) significantly improved milk yield in the whole lactation period, while short-term (5 days) administration did not change the milk production. The mechanisms underlying the improvement of lactation performance warrant further study.

## 5. Application of *S. baicalensis* in Disease Prevention and Treatment

Medicinal herbal preparations are mainly natural plant extracts, which can not only be used as feed ingredients but also have significant anti-parasitic, antibacterial, anti-viral, and other effects due to the effective bioactive compounds. Thus, they are of great value in the prevention and treatment of animal diseases.

Liao et al. [105] studied the therapeutic effect of baicalein injection on artificially infected swine edema disease in weaned piglets aged 28–35 days. Intraperitoneal injection of *Vibrio cholerae* O139 bacteria suspension was used to establish the swine edema disease model of artificially infected pigs, and different doses of *S. baicalensis* extract were injected for the treatment of the disease. It was found that the doses of 0.2 and 0.4 mL *S. baicalensis* extract/kg body weight significantly increased the weight gain rate of the experimental animals. The weight gain rate and total effective rate were 96% and 90%, respectively. This indicates that the injection of *S. baicalensis* extract could effectively treat the edema disease of artificially infected pigs.

Baicalin can effectively inhibit *Mycoplasma gallisepticum* induced immune deficiency and attenuate inflammatory response and apoptosis in the chicken bursa of Fabricius. Baicalin attenuates the levels of pro-inflammatory factors, and suppresses NF-κB expression at the protein and miRNA levels, alleviating reduction in *M. gallisepticum* induced CD8+ cells and bacterial burden in the bursa [121]. In addition, treatment with baicalin (450 mg/kg BW for 5 days) could effectively alleviate the extent of lesions in lung and tracheal tissues, alveolar space, and mucosal layer thickening were restored, cilia were gradually restored, and the IL-17 signaling pathway-related genes were significantly reduced. The activities of cytokines and chemokines (CXCL1, CXCL2, MMP1, GMCSF, and MUC5AC) were decreased significantly. Baicalin was able to effectively treat co-infection caused by *M. gallinarum* and *E. coli* [122]. Baicalin can resolve intestinal dysbacteriosis caused by H9N2 subtype avian influenza infection by modulating lactic acid bacteria, thus preventing the high mortality caused by the secondary infection of Escherichia. Baicalin had beneficial effects for clinical prevention and control of H9N2 Subtype Avian infection and secondary bacterial infections and inflammation by inhibiting the loss of intestinal structure and improving antioxidant capacity, affecting blood biochemical indexes, and inhibiting the production of inflammatory factors [123]. Baicalin effectively treats polyserositis caused by *Glaesserella parasitis* infection by alleviating the downregulation of mRNA for tight junctions, preventing the abnormalities, and maintaining the integrity of tight junctions, and is a potential natural medicine for the prevention and treatment of *G. parasitis* [124].

Baicalin showed significant antibacterial activity against *E. coli* in vitro. In a study by Zhao et al. [36], the minimum inhibitory concentration of baicalin against *E. coli* isolated from mastitis in dairy cattle was 4000 µg/mL, and antimicrobials such as streptomycin, ciprofloxacin, and ampicillin had synergistic effects in combination with baicalin. The combinations could significantly increase the susceptibility to *E. coli*. Therefore, baicalin may be used as an antimicrobial agent alone or in combination with antibiotics to treat *E. coli* caused mastitis in dairy cows. It was found that panton-valentine leucocidin (PVL)-killing interleukin produced by *S. aureus* was one of the causes of dairy cow mastitis. Apoptosis and necrosis of bovine mammary epithelial cells were associated with PVL, while baicalin could inhibit interleukin-producing *S. aureus* and consequently reduce apoptosis. Thus, baicalin has great potential for use in the treatment of mastitis caused by *S. aureus* [125]. High-yielding dairy cows are prone to oxidative reactions which are closely associated with inflammation. Baicalin has considerable anti-inflammatory and antioxidant effects on LPS-induced inflammatory damage of mammary epithelial cells in dairy cows. It can be used to prevent oxidative metabolic disorders in dairy cows and has been effectively applied in clinical practice [69,126].

Baicalin is widely used to treat viral diseases such as bovine viral diarrhea and enteritis [127]. Previous studies have shown that the cure rate of the decoction of *S. baicalensis* in conjunction with *Fructus gardeniae* on bovine diarrhea was 95%, and the cure rate in vivo was 75% [128]. In addition, the decoction of *S. baicalensis* in conjunction with *F. gardeniae* can directly inhibit the bovine viral diarrhea virus in vitro [129]. Lv et al. [106] showed that oral administration of baicalin at medium and low doses (850 and 425 mg/d for 5 days) could effectively treat piglet diarrhea and found that baicalin could inhibit inflammatory exudation and reduce the release of inflammatory cytokines, which might be one of the mechanisms of its therapeutic effect. Baicalin showed potent activity against Newcastle disease virus and direct killing effect against Newcastle disease, capable of inhibiting the infection of chicken embryo fibroblasts and blocking the intracellular Newcastle disease virus, and has the potential to be used as a pharmaceutical ingredient [130].

Baicalin had inhibitory effects on duck hepatitis virus (duck hepatitis A virus type 1, DHAV-1). An in vitro mechanistic study revealed that baicalin inhibited the propagation of DHAV-1 by interfering with the viral replication and release of the virus. An in vivo mechanistic study showed that the antioxidant and immunological enhancement functions of baicalin played a crucial role in its therapeutic effects against duck viral hepatitis. Baicalin may serve as a potential agent for the treatment of duck viral hepatitis [131].

## 6. Conclusions

*S. baicalensis* contains a variety of active compounds with multiple biological functions, such as antioxidation and bacteriostatic and anti-inflammatory effects. Thus, it has extensive potential in the production and health of food animals. At present, *S. baicalensis* is sometimes used in animal husbandry production in the form of composite preparations. The chemical composition of the composite preparations is complicated, which makes it challenging to elucidate mechanisms of actions and results in unstable effects. Therefore, it is particularly important to study monomer compounds present in medicinal herbs. Currently, most studies on the biological activity and pharmacological effects of baicalin, baicalein, and other extracts of *S. baicalensis* are conducted with laboratory animals and few studies have been performed in animal production and clinical application. There is an urgent need to strengthen the research and application of baicalin and other herbal active compounds in clinical practice. The substitution of antibiotics by natural herbs and their active compounds has become a trend in the future research and development of animal feed, with great potential in practice.

## Figures and Tables

**Table 1:** The biological functions and application of *Scutellaria baicalensis* and its active compounds.

Type of Product	Active Components	Biological Functions	Application	Target	References
Active compound	Baicalin	Antioxidation	Thymus protection	Chicken	[23,24,25]
		Antipyretic effect	Reducing mastitis incidence	Dairy cow, rat	[26,27,28,29]
		Analgesic effect	-	Rat	[30,31,32]
		Anti-inflammation	Pyemiapyaemia Cardiovascular disease ^1^	Human, mouse	[33] [34]
		Antibacterial effect	*Escherichia coli*	Dairy cow	[35,36,37,38]
		Antitumor activity	Pancreatic cancer ^1^ Liver cancer ^1^ Colon cancer ^1^ Leukemia ^1^	Human	[39] [40] [41] [42]
	Baicalein	Antioxidation	Protection against H_2_O_2_-induced oxidative injury of neuronal cells	Human	[19] [23,43,44,45]
			Heart protection	Chicken	
		Anti-inflammation	Treatment of skin diseases	Mouse	[46]
		Antimicrobial effect	*Staphylococcus aureus* and *E.coli*	Animals	[47,48]
			Candida		[49,50]
		Antitumor	Induction of tumor cell death	Human	[51]
	Wogonin	Cartilage protection	Treatment of arthritis ^1^	Mouse	[52]
		Anti-inflammation	Treatment of skin diseases	Animals	[53]
		Antivirus	Treatment of avian influenza	Chicken	[54]
Extract	*S. baicalensis* extract	Antibacterial effect	*Klebsiella pneumoniae* and *Pseudomonas aeruginosa*	-	[55,56]
		Antivirus	Avian infectious laryngotracheitis, tick borne encephalitis ^1^	Animals	[57,58]
Root	*S. baicalensis*	Antiallergic effect	Treatment of pruritus	Animals	[59,60,61,62,63,64,65]
		Inhibition of angiogenesis	Treatment of cancer ^1^	Human	[66]

^1^ Indicates potential in practical application.

**Table 2:** The main effects of *Scutellaria baicalensis* on animals.

Type of Animal	Age of Animals	Source	Dose or Concentration	Main Effects	References
Broilers	7–42 d	Baicalein	100–200 mg/kg diet	Improve growth performance, immunity, and antioxidant activity	[93]
Broilers	1–42 d	*S. baicalensis* roots	5, 10, 15 g/kg diet	Increase broiler body weight and feed conversion efficiency	[94]
Turkey hens	42–63 d	*S. baicalensis* extract	0.009, 0.018, 0.036 mL/kg BW ^1^	Alter the contents of sodium, potassium, calcium, magnesium, copper, zinc, and iron in plasma and affect meat quality	[95]
Laying hens	-	*S. baicalensis* extract	5 g/kg diet	Increase egg weight, decrease microbial content in the cecum, reduce the amount of propylene glycol in eggs and delay lipid oxidation in eggs	[96]
Chicken	9–24 d	*Curcuma longa* and *S. baicalensis* extracts	2 g/kg diet	Reduce intestinal inflammation and improve production performance	[97]
Broilers	1–42 d	*S. baicalensis* and *Lonicera japonica* extracts	500 mg/kg diet	Improve growth performance, promote the development of immune organs, and improve the antioxidant function	[98]
Broilers	1–49 d	Baicalin	20 mg/kg diet	Improve growth performance, blood parameters, nutrient digestibility, and meat quality	[99]
Weaned piglets	28–56 d	Fermented *Scutellaria*	1.5 mg/kg diet	Enhance appetite, increase average daily intake, reduce feed-to-weight ratio and diarrhea rate, and improve the feed reward	[100]
Weaned piglets	-	*S. baicalensis* extract	-	Improve growth performance and manipulate intestinal microflora	[101,102,103,104]
Weaned piglets	28–35 d	Baicalin	10 mg/kg BW, 1 time/d, 5 d; intramuscular injection	Prevent swine edema disease	[105]
Piglets	5–25 d	Baicalin	212.5 mg/time, 2 times/d, 5 d; oral administration	Prevent piglet diarrhea	[106]
Pregnant and lactating sows	-	Mixed herbs containing *S. baicalensis*	-	Decrease weight loss and improve litter performance	[107]
Dairy cows	-	*S. baicalensis* extract	100 g/kg diet	Reduce incidence of mastitis and improve milk yield	[26]

^1^ BW = body weight.
